# Human-introduced long-term traditions in wild redfronted lemurs?

**DOI:** 10.1007/s10071-013-0636-9

**Published:** 2013-05-14

**Authors:** Anna Viktoria Schnoell, Marie T. Dittmann, Claudia Fichtel

**Affiliations:** 1 Behavioral Ecology and Sociobiology Unit, German Primate Center, Kellnerweg 4, 37077 Göttingen, Germany; 2 Courant Research Center “Evolution of Social Behavior”, University of Göttingen, Göttingen, Germany; 3 Group of Animal Nutrition, Institute of Agricultural Sciences, ETH Zürich, Universitätsstrasse 2, 8092 Zurich, Switzerland

**Keywords:** Stability of traditions, Social learning, Scrounging, Long-term study, Wild lemurs, *Eulemur rufifrons*

## Abstract

Behavioural traditions have only been described for a small subset of species, and the factors responsible for the maintenance of traditions over time are unclear. Redfronted lemurs are known to learn socially but traditions have not been described in the wild. We conducted a social diffusion experiment over three experimental years with artificial feeding boxes that could be opened in two different ways (pushing or pulling a door). Six out of 14 individuals that participated in at least 2 years exhibited a stable preference: five lemurs maintained a pull and one lemur a push preference, suggesting that habit formation and reinforcement learning may have lead to preferences over time. The remaining individuals exhibited fluctuating preferences and switched between showing a preference or no preference, but never switched between preferences. This instability might have been due to the low level of difficulty and/or the low object specificity of the task. The majority of lemurs additionally scrounged. Scrounging was not influenced by age, sex or success in manipulating the boxes. Thus, redfronted lemurs appear to use the two techniques flexibly but also scrounged opportunistically to get access to the rewards, indicating that traditions might be stabilized by multiple factors.

## Introduction

Decades of experimental work, conducted in captivity as well as in the field, revealed that many animals are able to learn socially or that they at least possess the ability to use the social information provided by other individuals in the learning process (Reader and Hager 2011). Many species ranging from insects to mammals, including solitary reptiles (Wilkinson et al. 2010), are able to learn socially (Galef and Laland 2005; Leaderbeater and Chittka 2007; Laland et al. 2012). Although social learning seems to be widely spread in the animal kingdom, behavioural traditions have been documented in only a small subset of species. A tradition is a “distinctive behaviour pattern shared by two or more individuals in a social unit, which persists over time and that new practitioners acquire in part through socially aided learning” (Fragaszy and Perry 2003). Traditions have been documented in the wild in primates (Kawai 1965; Whiten et al. 1999; Perry et al. 2003; van Schaik et al. 2003; Perry 2009; Santorelli et al. 2011), cetaceans (Rendell and Whitehead 2001; Krützen et al. 2005) and other mammals (Thornton et al. 2010), birds (Hunt and Gray 2003; Berg et al. 2012) and fish (Helfman and Schultz 1984; Warner 1988).

Traditions are classified as one of the three different stages of diffusion of new behavioural pattern within groups (Huffman and Quiatt 1986; Huffman and Hirata 2003): The first stage of diffusion is social transmission. It is an incident of social learning that leads to the diffusion of a new behaviour within groups. Traditions represent the second stage, in which the behaviour has already spread and further diffusion at this stage is mainly constrained by birth rates. The third and last stage that can follow is the one of transformation, in which the behaviour gets modified in some way, to make it, for example, more efficient. Several factors have been suggested to promote and maintain traditions over time. Behavioural patterns might be more persistent if switching between alternative behaviours is not beneficial (Thornton and Clutton-Brock 2011). For example, the stability of foraging traditions is favoured when exploration of novel food items is linked to the risk of eating incompatible and/or poisonous food, or when searching new feeding routes increases the risk of predation by leaving the safety of the social group (Thornton and Clutton-Brock 2011). However, if an already acquired technique is more costly than an alternative technique, and if the difference of costs between the two techniques exceeds a certain threshold, animals might benefit from switching between behaviours (Thornton and Clutton-Brock 2011). Costs in the form of extensive time and effort, which individuals have to invest to acquire a certain skill can also influence the stability of foraging traditions; if the skill is difficult and time-consuming to obtain, it might be beneficial to maintain it, even if the alternative technique could be as rewarding (Thornton and Clutton-Brock 2011). Moreover, additional costs caused by conspecifics via scrounging, that is, getting access to a reward by taking advantage of the actions of other individuals, may also promote the instability of foraging traditions. Scrounging is a behaviour that might impose costs on the victims because they alone have to invest energy to obtain a reward, but then have to share it with others (McCormack et al. 2007).

High levels of individual conservatism, that is, the tendency to keep a once learned technique over time, seem to favour the stability of behavioural patterns or traditions because the behaviours can simply become habitual (Marshall-Pescini and Whiten 2008). A response habit is defined as an action that gets repeated by an animal because it was rewarded in the past (Pesendorfer et al. 2009; Crast et al. 2010). It has been suggested to be the stabilizing mechanism of the formation of a tradition in a long-term study in captive capuchins (*Cebus apella*: Crast et al. 2010) and in a short-term study of wild common marmosets (*Callithrix jacchus*: Pesendorfer et al. 2009).

The lack of social learning mechanisms that allow copying others in high fidelity, such as imitation, have been suggested to negatively influence the stability of traditions (Tomasello 1994). However, up to this date, imitation has been shown to be important for the propagation of behaviours only in captive animals (Whiten et al. 2004). Moreover, local enhancement has been suggested to be the dominant mechanism for the generation and maintenance of traditions in wild chimpanzees (*Pan troglodytes:* Inoune-Nakamura and Matsuzawa 1997) suggesting that a high level of fidelity in the copying might not be necessary for the stability of animal traditions (Caldwell and Millen 2009; Cladière and Sperber 2010).

Conformity, that is, the copying of the choice or behaviour of others even if the alternative is equally beneficial (Boyd and Richerdson 1985; Giraldeau et al. 2002), leads to higher homogeneity within groups or subgroups and therefore can have a stabilizing effect on traditions (Cladière and Sperber 2010). Conformity was proposed to explain the development of group preferences in an experiment in captive chimpanzees (Whiten et al. 2005). However, the rewarding character of behavioural traditions might be more crucial for the maintenance of traditions than the mechanism of diffusion (Galef 1995; Matthews et al. 2010). In fact, most of the behavioural traditions described for wild populations are rewarding, for instance the satisfaction of reaching and eating a food item (milk bottle opening in British tits (*Parus major*): Hinde and Fisher 1951; use of anvil and stone pounding tools in capuchins: Fragaszy et al. 2004) or the relief felt by eliminating parasites (leaf swallowing in chimpanzees: Huffman and Hirata 2004).

Although longevity of a behavioural variant is an important feature for a tradition (Whiten and van Schaik 2007), most experimental studies focused on the first stage of diffusion and examined whether different species are either able to learn socially, or whether the behaviour is transmitted within groups and/or how group preferences can develop. So far, only few experimental studies investigated the longevity of human-introduced traditions in animals (Cladière and Sperber 2010). For example, captive capuchin monkeys maintained a preference for a particular technique to open an artificial feeding box over 2 years (Crast et al. 2010), and wild vervet monkeys (*Chlorocebus aethiops*) maintained experimentally introduced food cleaning preferences over more than 1 year (van de Waal et al. 2012). In contrast, meerkats (*Suricata suricatta*), that initially shared the demonstrator’s preference to forage at one of the two land marks, did not maintain this preference over time and soon fed on both land marks equally often (Thornton and Malapert 2009). The inconsistency in these findings emphasises the importance to study the development of human-introduced traditions on a more longitudinal scale, because observed patterns during a short-term study can diminish over time.

Although arbitrary traditions have been shown to persist in captive groups of animals (Crast et al. 2010), it is unclear whether they do so in the wild where nutrition is limited, where average proximity between group members is probably lower and the risk of predation might be higher (Thornton and Clutton-Brock 2011). We therefore studied the longevity of human-introduced behavioural patterns in four social groups of wild redfronted lemurs (*Eulemur rufifrons*). This species is a suitable model as it exhibits a rather egalitarian social structure (Pereira et al. 1990; Pereira and Kappeler 1997; Ostner and Kappeler 2004), suitable for social learning (Coussi-Korbel and Fragaszy 1995). Moreover, it has been demonstrated that lemurs use socially aided learning in captive and wild settings (reviewed in Fichtel and Kappeler 2010; Kendal et al. 2010a; Fichtel and Kappeler 2011; Stoinski et al. 2011). In a previous study, we introduced an artificial feeding box that could be opened by two different techniques and showed that redfronted lemurs use social information to learn the feeding techniques and that individuals appeared to develop a group preference for one technique (Schnoell and Fichtel 2012). To examine whether redfronted lemurs maintain their individual and/or group preferences over time, we repeated these experiments over a period of 3 years, thereby gathering information on individual and group preferences for feeding techniques.

## Methods

### Study site and subjects

Experiments were conducted at the research station of the German Primate Center in Kirindy Forest, Western Madagascar (Kappeler and Fichtel 2012a). Study subjects were 42 redfronted lemurs (*Eulemur rufifrons*): 26 males and 16 females from four social groups (A, B, F and J). All subjects were individually marked with nylon collars and were well habituated to human presence (Kappeler and Fichtel 2012a, b). Experiments with feeding boxes were conducted in three consecutive years with 37 individuals in September–December 2009, with 40 individuals in August 2010 and with 32 individuals in May 2011 (Table 1). Four males switched between groups during the study period (MRot from group B to J, MNeg from B to A, MMyk from A to B and MGor from A to B).

**Table 1:** Number of participating individuals (≥3 task manipulations) and overall group size per study group and experimental year (year 1 = 2009, year 2 = 2010, year 3 = 2011)

Years	Group A			Group B			Group F			Group J		
	1	2	3	1	2	3	1	2	3	1	2	3
*N* participants	3	4	4	6	3	4	4	4	6	6	5	6
Group size	12	13	10	8	8	5	9	11	11	8	8	6

### Experimental setup and procedure

**Fig. 1:**
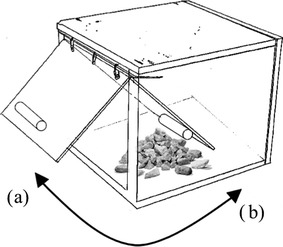
Experimental apparatus: the feeding box could be opened by either *a* pulling or *b* pushing a door to extract the reward from inside (artwork by AVS)

We presented redfronted lemurs wooden feeding boxes (Schnoell and Fichtel 2012; with a size of 16 × 20 × 20 cm; Fig. 1) that could either be opened by pulling or by pushing a semi-transparent door to get access to a food reward (several small pieces of orange or mango) inside the box. Feeding boxes were placed on an open spot on the forest floor to enable videotaping of all actions at the boxes. The experiment started when the first individual entered a 1-m radius around a box and ended either when the whole group left a 10-m radius around the boxes (for more detailed description see: Schnoell and Fichtel 2012) or after a maximum of 30 min (in 2010 and 2011). Each group was usually tested once a day between 07:00 and 17:00 h, and occasionally, groups were tested every second day or twice a day.

In the first year of the experiments (2009), two study groups (A and J) received training for one of the two opening techniques by constraining the box to a single functioning method over 7–10 sessions (group A: pulling; group J: pushing). Afterwards, they were confronted with unconstrained boxes in four additional test sessions. The two other study groups (B and F) did not receive any training and were tested with the unconstrained boxes over 14 sessions (Schnoell and Fichtel 2012). Preferences for one or the other technique were only determined for the last four sessions, during which all groups were confronted with unconstrained boxes.

In the second (2010) and third year (2011), we tested the four study groups with the same unconstrained feeding boxes in 12–14 sessions in 2010 (group A, B and J: 12 sessions; group F: 14 sessions) and 14 sessions in 2011. To increase the number of participating individuals, we presented redfronted lemurs in 2011 in the first 5 sessions with 3 boxes and in the following 7 sessions with 6 boxes.

The interval between the end of the first experiment and the beginning of the second experiment was 36 weeks (252 days) in 2009–2010 and 35 weeks (247 days) in 2010–2011.

### Data analyses

We analysed the number of successful as well as unsuccessful task manipulations and the technique used for each task manipulation from video recordings. Successful task manipulations were defined as moving the door and retrieving a reward, whereas an unsuccessful task manipulation was defined as moving the door but not gaining a reward. To compare the numbers of unsuccessful manipulations before the first successful manipulation over time, we included all individuals that managed to succeed in at least 2 years, regardless of how many actions they performed in total per year. Additionally, we recorded all scrounging events, that is, gaining access to the rewards by entering the feeding box which had been opened by another individual (the producer). We excluded events in which the producer left the box and a third individual scrounged from the first scrounger. Additionally, we recorded the technique the producer used to open the box.

To assess whether the number of unsuccessful task manipulations until the first success differed between years, we constructed a generalised linear mixed model (GLMM) by using the number of unsuccessful task manipulations until the first success as dependent variable, year as fixed factor and individual identity as random factor.

For the analysis of individual preferences for one or the other technique, we included only individuals that performed at least 6 actions at the boxes. Individual preferences for a feeding technique were analysed with a Binomial test. We also used a Binomial test to assess whether the number of individuals keeping a stable preference differed from the number of individuals with an unstable preference and to analyse whether the number of individuals exhibiting unstable preferences differed due to the technique they favoured in their first year. A stable preference was defined as keeping a preference for one technique from 1 year to the following year of participation. If individuals changed from 1 year to another, either by switching preferences or by switching from a preference to no preference or vice versa, we defined them as exhibiting an unstable preference. We constructed a GLMM to test whether a stable preference from 1 year to the other was influenced by sex, group membership, year (first: stability from 2009 to 2010, second: 2010 to 2011) or age class (juvenile–juvenile, juvenile–adult, adult–adult; juveniles <2.5 years, adults >2.5 years). Individual ID was used a random factor.

To assess whether the number of individuals performing both, scrounging and opening the box (producing) to gain rewards, differs from the number of individuals only scrounging or producing, we applied a χ^2^ test. To estimate whether the frequency of scrounging events is influenced by age, sex or success in handling the task (number of successful task manipulations), we used another GLMM. Age, sex and the number of successful task manipulations were used as fixed factors and individual identity nested in groups as random factors.

To assess whether individuals scrounged more often when other individuals opened the box by pushing or pulling the door, we used a GLMM. We used technique (pull or push) as fixed factor and individual identity as random factor. In order to analyse whether the stability of individual preferences is influenced by the frequency of being scrounged, we calculated a scrounging score for each individual in 2010 and 2011 (number of actions in which other individuals scrounged by the total number of actions). We calculated a GLMM by using stability as response variable, scrounging scores of 2010 and 2011 as fixed factor and individual identity as random factor. All GLMMs were fitted in R (R Development Core Team 2010), using the R package lme4 (Bates and Maechler 2010). The significance of the full model as compared to the null model (comprising only the intercept and the random effect) was established using a likelihood ratio test (R function Anova with argument test set to “χ^2^”). *P* values for the individual effects were based on Markov Chain Monte Carlo sampling (Baayen 2008) of the R package language R (Baayen 2010). Binomial tests and χ^2^ tests were conducted in IBM SPSS 20 (SPSS Inc., Chicago, IL, USA).

## Results

### Success in manipulating the box over the three experimental years

Thirty-two out of 42 members of the 4 study groups manipulated the feeding boxes (overall participation rate of 76.2 %). Twenty-five individuals performed successful manipulations in at least 1 year. Eighteen individuals opened the boxes successfully in 2009. Fifteen individuals managed to retrieve rewards in 2010, and 2 of these subjects did so for the first time. In 2011, 18 individuals conducted successful task manipulations; and 5 of these 18 individuals manipulated the boxes successfully for the first time. On average, redfronted lemurs conducted 54.4 ± 49.9 (mean ± SD) successful task manipulations in 2009, 40.1 ± 52.1 in 2010 and 59.7 ± 38.8 in 2011.

**Fig. 2:**
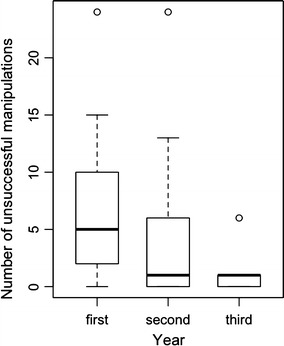
Number of unsuccessful task manipulations until the first success for individuals that learned the novel behaviours and participated in at least 2 years (1st year: *n* = 15 individuals; 2nd year: *n* = 15 individuals; 3rd year: *n* = 11 individuals; *boxplots* indicate median, upper and lower quartiles, outliers are indicated as *small dots*)

Fifteen subjects manipulated the feeding boxes successfully in at least 2 years of the experiments. In the first year of experiment, 13 % of 15 individuals were able to open the box right away, and 1 individual needed only 1 unsuccessful task manipulation before its first successful manipulation. The remaining 80 % needed between 2 and 24 trials before their first successful manipulation. In the second year of participation, 40 % of 15 individuals were able to open the boxes immediately with success, 13 % performed only 1 unsuccessful task manipulation, whereas 47 % needed between 2 and 24 trials until the first success. In the third year of participation, 45 % of 11 individuals were able to successfully open the box immediately, 45 % needed only 1 unsuccessful manipulation and 1 individual underwent 6 trials before the first success. The lemurs needed fewer unsuccessful task manipulations until the first successful one in the third year of participation compared to the first year of participation (Fig. 2; Table 3a; GLMM: χ^2^ = 6.08, *P* = 0.048).

### Preferences for opening techniques over time

Twenty-two out of 32 individuals performed ≥6 task manipulations and were therefore included in this analysis (2009: *N* = 15 individuals; 2010: *N* = 15 individuals; 2011: *N* = 17 individuals). Eight individuals participated in one single year, 3 individuals in two years and 11 individuals conducted actions in all 3 years (Table 2). On average, they conducted 32.3 ± 22.1 (mean ± SD) task manipulations (successful and unsuccessful) in 2009, 60.4 ± 47.6 in 2010 and 92.1 ± 52.1 in 2011.

**Table 2:** Preferences of subjects that performed ≥6 actions per year and corresponding *P* value of the binomial test

Group	Individual	Preference 2009	*P* value	Preference 2010	*P* value	Preference 2011	*P* value
A	FCor	Pull	<0.01	Pull	<0.01	Pull	<0.01
A	MKor	Pull	<0.01	Pull	<0.01	Pull	<0.01
B	FSip	Pull	<0.01	Pull	<0.01	Pull	<0.01
B	FBor	Pull	<0.01	Pull	<0.01	Pull	<0.01
F	MCas	Pull	<0.01	Pull	<0.01	Pull	<0.01
F	FMont	Push	0.04	No preference	0.29	Push	<0.01
F	MTri	Push	<0.01	No preference	0.21	Push	<0.01
J	FGeo	Push	<0.01	No preference	0.51	Push	<0.01
J	FMal	Push	<0.01	No preference	1.00	No preference	0.05
J	MKaz	Pull	<0.01	No preference	0.17	No preference	0.55
F	FLuc	No preference	1.00	Pull	<0.01	No preference	<0.01
J	MUsb	Push	<0.01	Push	<0.01	*No data*	
J	FCam	Push	<0.01	*No data*		No preference	0.24
A	MSky	*No data*		No preference	0.29	Pull	<0.01
A	MMil	*No data*		No preference	1.00	*No data*	
B	MLab	*No data*		No preference	0.31	*No data*	
B	MPan	Pull	0.01	*No data*		*No data*	
B, J	MRot^a^	*No data*		*No data*		Pull	<0.01
J	FMol	Push	<0.01	*No data*		*No data*	
A, B	MMyk^a^	*No data*		*No data*		Pull	<0.01
F	FAng	*No data*		*No data*		Push	<0.01
J	FCol	*No data*		*No data*		Pull	0.01

Acronyms indicate sex (1 letter) and name (2–4 letters)

*no data* = individual did not participate or did not conduct ≥6 actions

^a^Individuals that changed groups during the years

Individual preferences were rather unstable (Table 2). Eight out of the 14 individuals participating in 2 or 3 years switched between a preference for one technique and no preference. However, 6 individuals showed a stable preference (AFCor, AMKor, BFSip, BFBor, FMCas, JMUsb; acronyms: (1) letter = group, (2) letter = sex and (3)–(5) letters = name): 5 individuals kept a preference for pulling and 1 individual a preference for pushing. There was no difference between the number of individuals exhibiting a stable or an unstable preference (Binomial test: *N* = 14, *P* = 0.79). Individuals showing a preference for pushing in their first year of participation (*N* = 5) did not switch more often between preference and no preference than individuals exhibiting a preference for pulling (*N* = 1; Binomial test: *N* = 6, *P* = 0.22). Interestingly, not a single individual switched between preferences for the two techniques. The probability of exhibiting a stable preference was not influenced by sex, age, group membership or year (Table 3b, GLMM: χ^2^ = 9.16, *P* = 0.242). Eight individuals participated only in 1 year so that no preference over the years could be identified.

**Table 3:** Parameter estimated for the general linear mixed models (GLMM) (a) on the difference in the number of unsuccessful task manipulations until first success over the years, (b) on effects of group membership, age, sex and year on the stability of individual preferences, (c) on the difference in the number of scrounging events between individuals of different age and different sex and that performed different numbers of successful actions, (d) on the difference of task manipulation being scrounging between the two techniques and (e) on the difference in stability of individual preferences between individuals with different scrounging scores

Model	Response variable	Random factors	Fixed factors	Estimate	SE	*P* value
(a) GLMM	Number of unsuccessful task manipulations until first success	Individual identity	Intercept	6.73	1.46	<0.001
			2nd year	−2.67	2.06	NS
			3rd year	−2.24	2.24	0.015
(b) GLMM	Stability	Individual identity	Intercept	10.97	156.4	NS
			Juvenile–adult	0.37	244.7	NS
			Juvenile–juvenile	0.88	230.1	NS
			Sex	1.15	1.55.6	NS
			Group B	18.30	1,228,000	NS
			Group F	−24.25	234.0	NS
			Group J	−24.11	67.39	NS
			Year	0.34	199.3	NS
(c) GLMM	Number of scrounging actions	Individual identity	Intercept	2.99	0.89	NS
			Juveniles	−0.12	0.61	NS
			Females	0.41	0.26	NS
			Males	0.03	0.89	NS
			Success	0.01	0.76	NS
(d) GLMM	Scrounging (yes, no)	Individual identity	Intercept	−2.67	0.15	<0.001
			Method	0.28	0.14	0.041
(e) GLMM	Stability	Individual identity and group	Intercept	−11.17	20.12	NS
			Scrounging score	2.34	94.6	NS

*NS* not significant

**Fig. 3:**
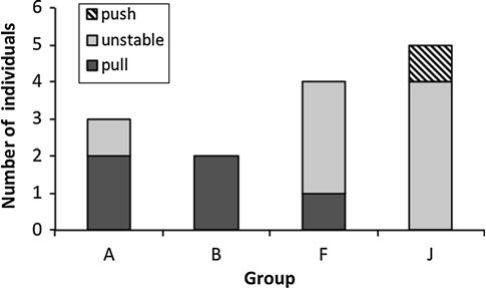
Number of individuals participating at least 2 years in the experiments and exhibiting a stable preference for pulling or pushing or switching between a preference and no preference

On the group level, 2 individuals each of groups A and B showed a stable preference for the pulling technique over the 3 years, and only 1 individual in group A switched between a preference and no preference (Fig. 3). In group F and J, however, 7 individuals showed unstable preferences, and in each group, only 1 individual exhibited a stable preference for one of the techniques (Fig. 3). Although the sample size is too small for statistical analysis, individuals of former pull group A and former open group B tended to be more stable in their preferences (80 % of individuals) than individuals of former open group F and former push group J (22 % of individuals).

### Scrounging

Individuals scrounged in 9.1 % of 4,079 task manipulations. Scrounging occurred in all 4 study groups (group A: *N* = 42 events; group J: *N* = 121 events; group B: *N* = 62 events; group F: *N* = 147 events) and in all 3 years (2009: *N* = 166 events; 2010: *N* = 58 events; 2011: *N* = 148 events). During one scrounging event, the number of scroungers varied between 1 and 3 individuals (one scrounger: *N* = 334 events; two scroungers: *N* = 37 events; three scroungers: *N* = 1 events).

Twenty-two individuals performed both successful task manipulations as well as scrounging actions at the feeding boxes. Two individuals only scrounged, 3 individuals never scrounged and 5 individuals neither succeeded nor scrounged. The majority of individuals used both tactics (producing and scrounging) to get access to the rewards (χ^2^ test: χ^2^ = 10.3, *df* = 1, *P* = 0.005). Interestingly, the frequency of scrounging was not influenced by age, sex or by the number of successful box manipulations (Table 3c; GLMM: χ^2^ = 0.58, *P* = 0.965). Scrounging occurred more often when producers opened the feeding box by pushing (11.7 %, *N* = 179 out of 1,536 push actions) than by pulling the door (7.6 %, *N* = 193 out of 2,543 pull actions; Table 3d; GLMM: χ² = 3.937, *df* = 1, *P* = 0.047, *N* = 4,079 task manipulations). However, the probability of whether individuals exhibited a stable preference from 1 year to the other could not be explained by the ratio of scrounging events (Table 3e; GLMM: χ^2^ = 0.006, *P* = 0.938). Therefore, victims of scrounging did not respond to a higher risk of scrounging events by exhibiting less stable preferences.

## Discussion

The present study reveals that redfronted lemurs opened an artificial feeding box successfully more quickly over the years, indicating that they seem to remember the rewarding character of the box over time, although there were time intervals of 9 months in between the presentation of feeding boxes. However, on the population level, they did not maintain a preference for one or the other technique over the three consecutive years. Individual preferences for the pulling technique tended to be more stable than preferences for the pushing technique, but more individuals tended to switch between exhibiting a preference or no preference. Moreover, the majority of animals also scrounged, and scrounging occurred more often when individuals opened the boxes by using the less stable pushing technique. However, the stability of a preference was neither influenced by the frequency of being scrounged nor by age, sex or group membership. Interestingly, the frequency of scrounging was not influenced by age, sex or success in opening the boxes. Thus, most redfronted lemurs appear to use the two techniques to open the feeding boxes flexibly and also scrounged opportunistically to get access to rewards.

The formation of long-term traditions has been suggested to be influenced by the difficulty of the task or the costs of modifying an established, effective behaviour pattern (Gajdon et al. 2004; Hopper et al. 2007; Thornton and Clutton-Brock 2011). A behaviour, which is difficult to learn, might be discovered only by rare innovators and is unlikely to spread within groups by social learning (Gajdon et al. 2004; Thornton and Clutton-Brock 2011). However, if a behavioural trait is easy to learn, it can be discovered by (most) individuals by asocial learning (Hopper et al. 2007; Thornton and Malapert 2009). Although redfronted lemurs used social information to acquire the opening techniques (Schnoell and Fichtel 2012), they also discovered the alternative technique. Individuals might therefore have not faced high levels of costs when switching between the two techniques because they did not have to invest a lot of time and effort to acquire the alternative behaviour. Thus, the box manipulations in this study might not have been physically difficult enough to induce long-term preferences in redfronted lemurs.

Persistence of arbitrary traditions might also be influenced by whether the different behaviours are highly option-specific, so that a generalisation from one option of the task to the other is unlikely (Hoppitt et al. 2012). Since the two options to gain a reward in this study did not differ in their difficulty to learn (Schnoell and Fichtel 2012) and could be solved by manipulating the same door, the low level of option specificity of the task may account for the fluctuation in preferences.

Nevertheless, 6 individuals exhibited a stable feeding technique preference over the 3 years. Although the sample size is rather small, neither age, sex or group membership influenced the probability of exhibiting a preference. These preferences were presumably formed by a response habit, that is, by sticking to the first rewarded technique (Crast et al. 2010). Another characteristic of habit formation is an increase in speed and accuracy in responding towards a stimulus (Neal et al. 2006), which can lead to a reinforcement of the already learned behaviour (Pesendorfer et al. 2009; Matthews et al. 2010). The individuals in this study became more efficient in manipulating the feeding boxes over the years, supporting the notion that habit formation is a likely mechanism for the formation of preferences. This is in line with other studies showing that simple learning mechanisms can explain the spread of two different pine-nut-eating traditions from mothers to offspring in wild rats (*Rattus rattus:* Terkel 1996) and novel foraging techniques in wild meerkats (Thornton 2008; Hoppitt et al. 2012), or that habit formation in combination with social facilitation and stimulus enhancement is the main mechanisms leading to a human-introduced long-term tradition in captive capuchin monkeys (Crast et al. 2010).

In addition to accessing rewards by opening the box by themselves, most redfronted lemurs also scrounged. During a single scrounging event, up to three individuals could scrounge, creating costs for the individual opening the box. Interestingly, the majority of individuals did both scrounging and manipulating boxes and did not use either tactic exclusively. Although scrounging occurred more often when individuals opened the box by pushing than by pulling the door, it did not influence whether individuals exhibited a stable preference as for example a pull-preference to avoid scrounging. Moreover, the probability of scrounging was not influenced by sex or age, suggesting that in this socially tolerant society, all group members are able and tolerated to scrounge. Since there was no relationship between scrounging and success in manipulating the boxes, redfronted lemurs appear to get access to the rewards rather opportunistically by either manipulating the boxes or scrounging.

Experiments with feeding apparatuses that can be opened in two distinctive ways are a common procedure to test for social diffusion in captive as well as field settings (reviewed by Whiten and Mesoudi 2008 and Kendal et al. 2010b). In primates, only two studies have investigated the longevity of human-introduced traditions experimentally: one in a captive population of capuchin monkeys over a period of 2 years (Crast et al. 2010) and one study in a wild population of vervet monkeys over 1 year (van de Waal et al. 2012). Our study represents an investigation over a period of 3 years in a field setting. Interestingly, redfronted lemurs did not maintain an experimentally introduced tradition over time. The intermediate pattern of some individuals exhibiting a clear preference over the 3 years and other individuals showing fluctuating preferences between a preference and no preference but not switching between preferences might have been influenced by several factors such as the formation of a response habit in some individuals, the opportunistically use of scrounging, the low levels of difficulty and/or object specificity of the task. Our results emphasise the importance of long-term studies to get a reliable picture in the area of social learning and animal traditions and to improve our understanding of the factors causing or preventing the stability of behavioural patterns over time.

## References

[CR1] Baayen RH (2008). Analyzing linguisitc data. A practical introduction to statistics using R.

[CR2] Baayen RH (2010) Language R: data sets and functions with “analyzing linguistics data: a practical introduction to statistics”. R package version 1.0. Available at http://CRAN.R-project.org/package=languageR

[CR3] Bates D, Maechler M (2010) lm4: linear mixed-effects models using S4 classes. R package version 0.999375-33. Available at http://CRAN.R-project.org/package=lme4

[CR4] Berg KS, Delgado S, Cortopassi KA, Beissinger SR, Bradbury JW (2012). Vertical transmission of learned signatures in a wild parrot. Proc R Soc B.

[CR5] Boyd R, Richerdson PJ (1985). Culture and the evolutionary process.

[CR6] Caldwell CA, Millen AE (2009). Social learning mechanisms and cumulative cultural evolution: is imitation necessary?. Psychol Sci.

[CR7] Cladière N, Sperber D (2010). Imitation explains the propagation, not the stability of animal culture. Proc R Soc B.

[CR8] Coussi-Korbel S, Fragaszy DM (1995). On the relation between social dynamics and social learning. Anim Behav.

[CR9] Crast J, Hardy JM, Fragaszy D (2010). Inducing traditions in captive capuchin monkeys (*Cebus apella*). Anim Behav.

[CR10] Fichtel C, Kappeler PM, Kappeler PM, Silk J (2010). Human universals and primate symplesiomorphies: establishing the lemur baseline. Mind the gap: tracing the origins of human universals.

[CR11] Fichtel C, Kappeler PM (2011). Variation in the meaning of alarm calls in Verreaux’s and Coquerel’s sifakas (*Propithecus verreauxi, P. coquereli*). Int J Primatol.

[CR12] Fragaszy DM, Perry S (2003). The biology of traditions: models and evidence.

[CR13] Fragaszy DM, Izar P, Visalberghi E, Ottoni EB, de Oliveira MG (2004). Wild capuchins (*Cebus libidinosus*) use anvils and stone pounding tools. Am J Primatol.

[CR14] Gajdon G, Fijn N, Huber L (2004). Testing social learning in a wild mountain parrot, the kea (*Nestor notabilis*). Learn Behav.

[CR15] Galef BG (1995). Why behaviour patterns that animals learn socially are locally adaptive?. Anim Behav.

[CR16] Galef BG, Laland KN (2005). Social learning in animals: empirical studies and theoretical models. BioScience.

[CR17] Giraldeau LA, Valone TJ, Templeton JJ (2002). Potential disadvantages of using socially acquired information. Philos Trans R Soc B.

[CR18] Helfman GS, Schultz ET (1984). Social transmission of behavioural traditions in a coral reef fish. Anim Behav.

[CR19] Hinde RA, Fisher J (1951). Further observations on the opening of milk bottles by British birds. Brit Birds.

[CR20] Hopper LM, Spiteri A, Lambeth SP, Schapiro SJ, Horner V, Whiten A (2007). Experimental studies of traditions and underlying transmission processes in chimpanzees. Anim Behav.

[CR21] Hoppitt W, Samson J, Laland KN, Thornton A (2012). Identification of learning mechanisms in a wild meerkat population. PLoS One.

[CR22] Huffman MA, Hirata S, Fragaszy DM, Perry S (2003). Biology and ecology of primate behavioural tradition. The biology of traditions: models and evidence.

[CR23] Huffman MA, Hirata S (2004). An experimental study on leaf swallowing in captive chimpanzees-insights into the origin of a self-medicative behavior and the role of social learning. Primates.

[CR24] Huffman MA, Quiatt D (1986). Stone handling by Japanese macaques (*Macaca fuscata*): implications for tool use of stones. Primates.

[CR25] Hunt CR, Gray RD (2003). Diversification and cumulative evolution in new Caledonian crow tool manufacture. Proc R Soc B.

[CR26] Inoune-Nakamura N, Matsuzawa T (1997). Development of stone tool use by wild chimpanzees (*Pan troglodytes*). J Comp Psychol.

[CR27] Kappeler PM, Fichtel C, Kappeler PM, Watts DP (2012). A 15-year perspective on the social organization and life history of Sifaka in Kirindy Forest. Long-term field studies of primates.

[CR28] Kappeler PM, Fichtel C (2012). Female reproductive competition in *Eulemur rufifrons*: evidence and reproductive restraint in a plurally breeding Malagasy primate. Mol Ecol.

[CR29] Kawai M (1965). Newly-acquired pre-cultural behavior of the natural troop of Japanese monkeys on Koshima islet. Primates.

[CR30] Kendal RL, Custance D, Kendal JR, Vale G, Stoinski T, Rakotomalala NI, Rasaminanana H (2010). Evidence for social learning in wild lemurs (*Lemur catta*). Learn Behav.

[CR31] Kendal RL, Galef BG, van Schaik CP (2010). Social learning research outside the laboratory: how and why?. Learn Behav.

[CR32] Krützen M, Mann J, Heithaus MR, Connor RC, Bejder L, Sherwin WB (2005). Cultural transmission of tool use in bottlenose dolphins. Proc Natl Acad Sci.

[CR33] Laland KN, Atton N, Webster MM (2012). From fish to fashion: experimental and theoretical insights into the evolution of culture. Philos Trans R Soc B.

[CR34] Leaderbeater E, Chittka L (2007). Social learning in insects—from miniature brains to consensus building. Curr Biol.

[CR35] Marshall-Pescini S, Whiten A (2008). Chimpanzees (*Pan troglodytes*) and the question of cumulative culture: an experimental approach. Anim Cogn.

[CR36] Matthews LJ, Paukner A, Suomi SJ (2010). Can traditions emerge from the interaction of stimulus enhancement and reinforcement learning? An experimental model. Am Anthropol.

[CR37] McCormack JE, Jablonski PG, Brown JL (2007). Producer–scrounger roles and joining based on dominance in a free-living group of Mexican jay (*Aphelocoma ultramarine*). Behaviour.

[CR38] Neal DT, Wood W, Quinn JM (2006). Habits—a repeat performance. Curr Dir Psychol Sci.

[CR39] Ostner J, Kappeler PM (2004). Male life history and the unusual adult sex ratio in redfronted lemurs (*Eulemur fulvus rufus*) groups. Anim Behav.

[CR40] Pereira ME, Kappeler PM (1997). Divergent systems of agonistic behaviour in lemurid primates. Behaviour.

[CR41] Pereira ME, Kaufman R, Kappeler PM, Overdorff DJ (1990). Female dominance does not characterize all of the Lemuridae. Folia Primatol.

[CR42] Perry S (2009). Conformism in the food processing techniques of white-faced capuchin monkeys (*Cebus capucinus*). Anim Cogn.

[CR43] Perry S, Barker M, Fedigan L, Gros-Louis J, Jack K, MacKinnon KC, Manson JH, Pranger M, Pyle K, Rose L (2003). Social conventions in wild white-faced capuchin monkeys: evidence for traditions in a neotropical primate. Curr Anthropol.

[CR44] Pesendorfer MB, Gunhold T, Schiel A, Souto A, Huber L, Range F (2009). The maintenance of traditions in marmosets: individual habit, not social conformity? A field experiment. PloS One.

[CR45] R Development Core Team (2010). R: a language and environment for statistical computing.

[CR46] Reader SM, Hager Y (2011). The evolution of primate general and cultural intelligence. Philos Trans R Soc B.

[CR47] Rendell L, Whitehead H (2001). Culture in whales and dolphins. Behav Brain Sci.

[CR48] Santorelli CJ, Schaffner CM, Campbell CJ, Notman H, Pavelka MS, Weghorst JA, Aureli F (2011). Traditions in spider monkeys are biased towards the social domain. PLoS One.

[CR49] Schnoell AV, Fichtel C (2012). Wild redfronted lemurs (*Eulemur rufifrons*) use social information to learn new foraging techniques. Anim Cogn.

[CR50] Stoinski TS, Drayton LA, Price EE (2011). Evidence of social learning in black-and-white ruffed lemurs (*Varecia variegate*). Biol Lett.

[CR51] Terkel J, Heyes C, Galef BG (1996). Cultural transmission of feeding behavior in the black rat (*Rattus rattus*). Social learning in animals: the roots of culture.

[CR52] Thornton A (2008). Social learning about novel foods in young meerkats. Anim Behav.

[CR53] Thornton A, Clutton-Brock T (2011). Social learning and the development of individual and group behaviour in mammal societies. Philos Trans R Soc B.

[CR54] Thornton A, Malapert A (2009). The rise and fall of an arbitrary tradition: an experiment with wild meerkats. Proc Biol Sci.

[CR55] Thornton A, Samson J, Clutton-Brock T (2010). Multi-generational persistence of traditions in neighbouring meerkat groups. Proc R Soc B.

[CR56] Tomasello M, Wrangham RW, McGrew WC, de Waal FBM, Heltne PG (1994). The questions of chimpanzee cultures. Chimpanzee culture.

[CR57] van de Waal E, Krützen M, Hula J, Goudet J, Bshary R (2012). Similarity in food cleaning techniques within matrilines in wild vervet monkeys. PLoS One.

[CR58] van Schaik CP, Ancrenaz M, Borgen G, Galdikas B, Knott CD, Singleton I, Suzuki A, Utami SS, Merrill M (2003). Orangutan cultures and the evolution of material culture. Science.

[CR59] Warner RR (1988). Traditionality of mating sites preference in a coral reef fish. Nature.

[CR60] Whiten A, Mesoudi A (2008). Establishing an experimental science of culture: animal social diffusion experiments. Philos Trans R Soc B.

[CR61] Whiten A, van Schaik CP (2007). The evolution of animal ‘cultures’ and social intelligence. Philos Trans R Soc B.

[CR62] Whiten A, Goodall J, McGrew WC, Nishida T, Reynolds V, Sugiyama Y, Tutin CEG, Wrangham RW, Boesch C (1999). Cultures in chimpanzees. Nature.

[CR63] Whiten A, Horner V, Litchfield CA, Marshall-Pescini S (2004). How do apes ape?. Learn Behav.

[CR64] Whiten A, Horner V, de Waal FBM (2005). Conformity to cultural norms of tool use in chimpanzees. Nature.

[CR65] Wilkinson A, Kuenstner K, Mueller J, Huber L (2010). Social learning in a non-social reptile (*Geochelone carbonaria*). Biol Lett.

